# Quack Dentists

**Published:** 1849-07

**Authors:** 


### QUACK DENTISTS.

BY ————————.

On Monday morning, the 5th of February, '49, a stranger called on me, who represented himself as a citizen of Franklin county, Miss., who wished to purchase a small portion ot *gold leaf.* I informed him that I had none, that I did not use it; at the same time suspecting that he did not know what he wanted, I made free to inquire what use he wished to make of it. He informed me that he had a job of Dentistry on hand, but that he had not gold enough to finish it, and that I would very much oblige him, &c. But I was apprehensive that a man who did not know what he wanted, would in all probability be equally at fault in using it if he had it, so I made the most plausible excuse that I could find, for not being prepared to accommodate him. I then plied him with a few plain simple questions in regard to the field of his professional labors, the extent of his practice, &c., &c.

From his replies, I gathered the information that his principal occupation was that of a Cancer Doctor, that he had a remedy that was certain death on cancer, and all kinds of scrofulous diseases, that he only practiced Dentistry a little occasionally, when he happened to find a job, and leisure, from the more arduous duties of his higher profession; that he had met a man in the neighborhood who required his services in the Dental line, that he had not quite enough gold to fill his teeth, and that I would confer a favor on himself, and a blessing on his patient if I would supply his wants. But as I happened to differ in opinion with him, in regard to the latter part of that beneficent enterprise, I most cordially begged leave to decline the honor proposed, and so we parted company. Of the man or his performances, farther than what I gathered from the above described interview, I have no knowledge whatever, but from his appearance and conversation, I was disposed to regard him as some aspiring genius, whose ambition prompted him to seek his own advantage at the expense of his more simple and confiding neighbors.

The next case that I propose to notice, is that of a man who passed through this village last winter with a Dentifrice, which he recommended in the most extravagant terms. I do not know what it was, otherwise than, by the taste, smell, &c., but a sample that I submitted to these tests, gave the result as simple pulverized chalk, with perhaps some kind of perfume. The individual who showed me the specimen, had purchased it with the assurance that it would fasten a tooth that was just ready to drop out from absorption of the gums and processes; most of its immediate neighbors having been lost in that way already. But this patent, this almost miraculous remedy was warranted to make the loose tooth perfectly fast in the short space of twenty-four hours, and at the same time to heal up sound and well all the cavities that existed in the rest of the teeth, and all for the astonishingly low price of twenty-five cents. But after disposing of a number of bottles, the vender of this wonderful remedy was next morning no where to be found. Not one word need be said in regard to the character of one who would take such an advantage of the ignorant or afflicted. The next case to which I invite your notice, is that of a youth, the same, I presume, that was characterized in the last number of the Register as a *Steam Dentist.* I did not see him, but am credibly informed that he professes to be able to remove the nerve from an aching tooth without the least pain, (thus rendering it safe, serviceable, and comfortable for life,) after exposing it to the fumes of a pill, for a few seconds, which he prepared according to secret instructions.

When his services were called into requisition, he placed one of his pills on a common one-fourth pound store weight, heated to a moderate degree; over this he placed an inverted funnel, to the apex of which was attached a flexible tube, which conveyed the vapor into the cavity of the tooth. But his attempt on the steamboat was not the only one which proved a failure, for my informant assured me that he extirpated seven or eight nerves from one aching tooth, at as many different times, without procuring any sensible relief. I could give the name and residence, or rather the place where he "cum frum," but forbear out of regard to his relatives.

Permit me now to offer a few thoughts relative to the source or foundation of these evil practices. While some of the knowing ones might be disposed to ascribe them to the cupidity and duplicity of those who practice them, I am disposed to place them to the credit of the too generally prevalent ignorance and credulousness in reference to such matters, which serves as an occasion or influence to call into active exercise those propensities that might otherwise lie dormant in the breasts or skulls of those who are in many if not in all cases, both morally and mentally depraved, and thus they become the victim of circumstances, for which they are no more responsible than the vile insect that feeds on the decomposing carcass is responsible for the putrefaction that breeds him.

